# Fostering healthcare system sustainability through efficient practices: Can adopting biosimilars ease the financial burden of rheumatoid arthritis?

**DOI:** 10.12688/f1000research.156983.1

**Published:** 2024-10-04

**Authors:** Christos Ntais, Nikolaos Kontodimopoulos, John Fanourgiakis, Michael A. Talias

**Affiliations:** 1 Healthcare Management Program, School of Economics & Management, Open University of Cyprus, Nicosia, Cyprus; 2 Department of Economics and Sustainable Development, Harokopio University, Athens, Greece; 3 Healthcare Management Program, School of Social Sciences, Hellenic Open University, Patras, Greece; 4 Department of Management Science and Technology, Hellenic Mediterranean University, Agios Nikolaos, Crete, Greece

**Keywords:** biosimilars, biologics, budget impact analysis, cost-effectiveness analysis, pharmacoeconomics, rheumatoid arthritis

## Abstract

Immune-mediated inflammatory diseases like rheumatoid arthritis (RA) have been successfully treated using biologic disease-modifying antirheumatic drugs. These medications are not utilized as first-line treatment, in part because of their high cost, but they are frequently seen to be cost-effective for RA patient populations that do not respond adequately to conventional disease-modifying antirheumatic drugs. Moreover, not all RA patients who meet clinical eligibility criteria can access biologics, not even as second-line therapy. Recently, there has been an increasing interest in biosimilars that are highly comparable to their originator biologics in terms of efficacy and safety but generally come at a lower price. This review summarizes the potential role of biosimilars in reducing RA expenditure and increasing RA patient access to biologic therapies. As the global landscape for biosimilars continues to evolve, it is essential to consider the unique challenges and opportunities in different healthcare systems. By leveraging the potential of biosimilars, healthcare systems can improve RA management, ease its economic burden and ensure that patients have access to effective and affordable treatments. The future of RA treatment lies in the integration of biosimilars into clinical practice, offering hope for more sustainable and equitable healthcare systems.

## 1. Introduction

Rheumatoid Arthritis (RA) is an autoimmune condition characterized by persistent inflammation of the synovial joints, which can cause joint degeneration, functional disability and systemic complications. About 0.5-1% of people worldwide, most of whom are women, are affected by the disease, which frequently leads to severe disability and an increased need for healthcare resources. RA treatment has changed dramatically with the introduction of biologic treatments, which provide notable gains in disease control. ^1^ Hence, the high cost of biologic treatments, especially over an extended period of time, places a significant financial strain on healthcare systems around the world. ^2^

Biosimilars, i.e. biologically similar medicines, have become more and more affordable substitutes for original biologics (originators) when the patents of the latter expire. ^3^ A biosimilar product shares many quality, safety and efficacy characteristics with an approved reference product. It is anticipated that the introduction of biosimilars will lower drug costs, broaden access to biologic treatments and enhance the sustainability of healthcare systems. ^4^ This paper investigates the economic impact of use of biosimilars by RA patients, with an emphasis on the sustainability of healthcare systems.

## 2. Biologic treatments in RA

Biologic disease-modifying antirheumatic medications (bDMARDs) have become a mainstay in the treatment of moderate to severe RA, especially for patients who do not respond well to conventional synthetic DMARDs (csDMARDs). ^1^ These biologics, including indicatively interleukin-6 (IL-6) inhibitors (like tocilizumab) and tumor necrosis factor (TNF) inhibitors (like infliximab, etanercept and adalimumab), among others, have shown impressive success in reducing disease activity and improving patients’ quality of life. ^1^

However, healthcare systems encounter a substantial financial burden due to the high cost of bDMARDs, especially in settings with limited resources. The fact that RA is a chronic disease that requires permanent or extended treatment intensifies this financial burden even more. As a result, the need for and cost of biologic medicines are on the rise, posing a risk for the sustainability of healthcare systems.

## 3. Inequities in access to biologics

TNF inhibitor availability has enhanced RA patients’ quality of life and lessened the disease burden for society as a whole. ^5^ A number of studies have demonstrated that RA is more severe in poorer countries and that treatment access disparities may contribute to this finding. In addition to demonstrating a negative association between gross domestic product (GDP) and the average disease activity score (DAS28), the QUEST-RA study found that, in low-GDP countries, biologically treated RA patients had a significantly lower DAS28 than non-biologically treated patients. ^6^ This finding suggests that not all patients in low-GDP countries receive the necessary biologic treatment. Furthermore, data from the COMORA study demonstrated that the association between disease activity and socioeconomic position is evident both at individual and country level (measured as GDP). ^7^

According to Putrik et al., ^8, 9^ there are serious disparities in RA patients’ access to biologic treatments. Out of 46 European countries, 22% did not have any reimbursement for biologics available, whereas 59% presented annual treatment costs that exceeded the GDP per capita, up to a maximum of 11 times. The country’s socioeconomic status and RA health indicators were positively correlated with the number of approved and reimbursed biologics ^8^ and a more lenient prescribing policy. ^9^ Again closely related to GDP, accessibility was determined by availability, affordability and acceptability, which differed across countries. ^8, 9^ Another study in a real-world setting revealed that bDMARDs use in RA patients varied considerably across six central and eastern European countries. ^10^

## 4. The emergence of biosimilars in RA

Biosimilars are biologic medicines that, in terms of structure, mode of action and clinical efficacy, are very similar to their already approved originator biologics. Although biosimilars are not identical to originator biologics due to the fact that biologic manufacturing processes are complicated, the development of biosimilars must adhere to strict regulatory requirements that guarantee their quality, safety and efficacy.

With biosimilars entering the RA treatment armory, there is a big opportunity to cut healthcare expenses without sacrificing therapeutic outcomes. Biosimilars provide significant cost savings potential since they typically enter the market between 20 and 40 percent less expensive than their originator biologics. As the patents on some well-known biologics, including adalimumab, etanercept and infliximab, have expired, a number of biosimilars have been already approved and introduced into certain international markets ( Table 1).

**Table 1.** Biosimilars approved for RA treatment by the EMA and the FDA up to August 2024.

Originator	EMA approved			FDA approved		
	Biosimilar	Manufacturer	Approval date	Biosimilar	Manufacturer	Approval date
Adalimumab (Humira)	Amgevita	Amgen	2017/03	Amjevita	Amgen	2016/09
	Imraldi	Samsung Bioepis	2017/08	Cyltezo	Boehringer Ingelheim	2017/08
	Cyltezo	Boehringer Ingelheim	2017/11	Hyrimoz	Sandoz	2018/10
	Hyrimoz	Sandoz	2018/07	Hadlima	Samsung Bioepis	2019/07
	Hulio	Mylan	2018/09	Abrilada	Pfizer	2019/11
	Idacio	Fresenius Kabi	2019/04	Hulio	Mylan	2020/07
	Amsparity	Pfizer	2020/02	Yusimry	Coherus BioSciences	2021/12
	Yuflyma	Millmount Healthcare	2021/02	Idacio	Fresenius Kabi	2022/12
	Libmyris	Stada Arzneimittel	2021/11			
Etanercept (Enbrel)	Benepali	Samsung Bioepis	2016/01	Erelzi	Sandoz	2016/08
	Erelzi	Sandoz	2017/06	Eticovo	Samsung Bioepis	2019/04
	Nepexto	Mylan	2020/05			
Infliximab (Remicade)	Remsima	Celltrion	2013/09	Inflectra	Celltrion	2016/04
	Inflectra	Hospira	2013/09	Renflexis	Samsung Bioepis	2017/04
	Flixabi	Samsung Bioepis	2016/05	Ixifi	Pfizer	2017/12
	Zessly	Sandoz	2018/05	Avsola	Amgen	2019/12
Rituximab (Rituxan/MabThera)	Truxima	Celltrion	2017/02	Truxima	Celltrion	2019/12
	Rixathon	Sandoz	2017/06	Ruxience	Pfizer	2021/11
	Riximyo	Sandoz	2017/06	Riabni	Amgen	2022/06
	Ruxience	Pfizer	2020/04			

EMA: European Medicines Agency; FDA: U.S. Food and Drug Administration; RA: Rheumatoid Arthritis.

## 5. Economic implications of biosimilars adoption in RA

The emergence of biosimilars has provided a less costly option to biologics. The use of biosimilars in RA treatment poses an economic impact that may be examined from different point of view, including medication costs, the utilization of healthcare resources, patient outcomes and wider implications for the sustainability of healthcare systems.

### 5.1 Drug prices

The adoption of biosimilars has several direct and noticeable advantages, one of which being the lowering of originator biologics prices, which can result in significant cost savings when added up over the course of entire healthcare systems, especially in countries with high consumption of biologics. In the face of biosimilars competition, biologics may see price reductions that, depending on biosimilar uptake, might result in significant savings for payers, patients and society as a whole. Maksabedian Hernandez et al. ^11^ estimated how the introduction of biosimilars would affect originators prices and spending in the US. Changes in the average price of originators were primarily linked to biosimilar uptake. One year following exclusivity milestones, prices of originators with relatively high biosimilar uptake (>60%) dropped significantly (-21.2% to -59.3%), while originators with weaker biosimilar uptake (<10%) presented smaller price drops (-2.4% to -8.4%).

Manova et al. ^12^ found that the introduction of biosimilars to national markets resulted in a significant decrease in reimbursed originators prices paid by public funds, which may improve patients’ access to biologic therapies. This was determined by conducting a comparative price analysis of biologics for RA treatment among 17 EU countries. However, unlike commodity generics, biosimilars prices did not significantly decrease following their entrance on the market. In Poland, the price base of originators has decreased to comparable levels of treatment with biosimilars as a result of the introduction of etanercept and infliximab biosimilars. ^13^

Since 2006, Norway has operated a national tender system for biologics, in which companies submit bids for their products, with cost serving as the primary criterion for selection. ^14^ This applies to both new therapies and when switching therapies for medical reasons. ^15^ The uptake of biosimilars has been impacted by the greater competition brought about by this approach. The price of a biosimilar infliximab (CT-P13) was as much as 39% less than that of the originator when it was released in 2014, and dropped down to 69% less a year later. ^16^ Despite a significant rise in use, the overall cost has been reduced by approximately $80 million in a population of roughly 5.5 million people. Additionally, there has been a trend in Norway to begin bDMARD treatment sooner and at a lower level of disease activity. ^17^

### 5.2 Healthcare resource utilization

In addition to direct drug cost savings, the utilization of healthcare resources can be also broadly impacted by the adoption of biosimilars. For example, the lower cost of biosimilars may allow a biologic therapy to be used earlier or more widely in patients with RA, which could in turn result in improved disease control and fewer long-term complications. ^18^ This could lead to less hospital stays, surgeries and other expensive treatments linked to uncontrolled RA. ^19^

Additionally, the adoption of biosimilars might promote more economical prescribing patterns. For instance, in healthcare systems with cost-sharing mechanisms, prescribers would be more inclined to transfer patients to a less costly biosimilar if there is a substantial cost saving between the biosimilar and the originator. This can optimize the overall management of RA and increase the economic benefits of biosimilars.

It is crucial to note that the effect of biosimilars on the utilization of healthcare resources may differ depending on the particular healthcare system, the patient population treated and the treatment scheme used. For instance, compared to systems with more segmented or decentralized processes, the introduction of biosimilars may have a more noticeable impact on resource utilization in systems with strict cost-control measures or centralized drug procurement procedures.

### 5.3 Patient outcomes and treatment adherence

The way that the adoption of biosimilars has an economic impact also depends on treatment adherence and patient outcomes. Biosimilars are a viable choice for RA patients who have switched from originator biologics to biosimilars, as supported by several studies that demonstrate similar clinical outcomes. For instance, according to a study by Jørgensen et al., ^20^ patients who continued on originator infliximab and those who switched to its biosimilar CT-P13 did not significantly vary in terms of disease activity, adverse events or treatment discontinuation rates.

By extending national reimbursement criteria, the cost savings from the use of biosimilars can enhance patient access. Improved clinical outcomes could be a benefit for both patients and healthcare professionals. For instance, RA patients with early or moderate disease have been shown to benefit from access to biologics, in terms of clinical and functional outcomes. ^21, 22^

Biosimilars have the potential to modify rheumatologists’ clinical practice in addition to treating more patients. Prior experience with biosimilar filgrastim has shown that physicians in several European centers have been urged to utilize this biologic medication as primary prophylaxis of febrile neutropenia in the context of chemotherapy rather than secondary prophylaxis due to its affordability. ^23^ In the same way, rheumatologists might be less reluctant to start biological therapy if a patient doesn’t respond well enough to csDMARDs. This is crucial for patients with poor prognostic factors, who gain the most from early bDMARD treatment.

On the other hand, treatment adherence and patient outcomes may be impacted by how patient view and accept biosimilars. Non-adherence or therapy discontinuation may result from concerns about switching, such as worries about decreased efficacy, higher side effects or the stigma associated with getting a “cheaper” option. It is imperative to tackle these issues by means of patient education, collaborative decision-making and open communication to guarantee the successful use of biosimilars and to optimize their economic benefits.

### 5.4 Broader implications for healthcare system sustainability

The adoption of biosimilars has wider economic ramifications than only effects on specific patients or healthcare professionals. The launch of biosimilars can ease financial strains, enhance access to biologic medicines and encourage competition in the pharmaceutical industry – all of which can help to the overall sustainability of healthcare systems. ^24^

The sustainability of healthcare systems is increasingly at risk, as global healthcare expenditure continues to climb due to factors like aging populations, rising rates of chronic diseases and the advent of expensive treatments. As biologics are among the most expensive medicines within modern healthcare systems, biosimilars provide a strategic opportunity to address some of these issues. The money saved by using biosimilars can be put back into the healthcare system in other ways, such as paying for R&D, increasing access to care or upgrading infrastructure.

Furthermore, by expanding access to biologic medicines for patients in low- and middle-income countries (LMICs) or underserved populations within high-income countries, the utilization of biosimilars can improve equity in healthcare. Because originator biologics are expensive and not available in many LMICs, many RA patients are left without access to effective treatment. Since they are less expensive, biosimilars have the potential to narrow this gap and improve patient outcomes in certain situations. Moreover, the launch of biosimilars may increase competition in the pharmaceutical industry and result in additional price reductions both for originator biologics and for biosimilars.

## 6. Challenges and barriers to biosimilar adoption

Even though biosimilars come with quite obvious economic benefits, a number of challenges and barriers prevent their widespread adoption and the turn of all their potential into value. Various reasons may account for low uptake of biosimilars, including regulatory, clinical and market-related barriers, the attitudes of physicians and patients to the use of these drugs and the reimbursement conditions in different countries.

### 6.1 Regulatory and approval processes

The frameworks regulating biosimilars vary broadly among regions and countries. Some jurisdictions have more complicated or stringent authorization procedures than others. ^25^ Although the U.S. Food and Drug Administration (FDA) and the European Medicines Agency (EMA) have set forth robust specifications and rules for the approval or authorization of biosimilars, other regions may have less defined procedures, which could give rise to delays in the introduction of biosimilars or fragmentation of the market.

Another major obstacle may also be the need for clinical trials to prove biosimilarity, especially for start-ups or small biopharmaceutical firms. Due to the time and cost that these trials entail, fewer biosimilars may reach the market, which would lessen competition, limit the possibility of price reductions for originators and cost savings in general.

### 6.2 Physician and patient acceptance

The successful adoption of biosimilars greatly depends on their acceptability by physicians and patients. Healthcare professionals and patients may continue to have concerns regarding the safety, efficacy and immunogenicity of biosimilars in comparison to originator biologics, even when regulatory bodies demand evidence of biosimilarity. ^26^

Concerns about possible variations in clinical outcomes or patient resistance may make physicians hesitant to switch stable patients from an originator biologic to a biosimilar. Likewise, patients who have had positive experiences with the originator biologic may be reluctant to move to a biosimilar if they believe it to be a lower quality or less effective alternative.

Another problem with switching is loss of efficacy or the onset of an adverse event that cannot be attributed to the drug’s recognized pharmacology. This phenomenon, referred to as the “nocebo effect”, is not unique to biosimilars and can happen anytime a patient has a negative opinion of a medication. ^27^ Patients may decide to return to the originator product as a result of this, decreasing drug adherence. ^28^ Indeed, higher dropout rates in open-label relative to double-blind studies have demonstrated a potential nocebo effect in biosimilars research. ^29^ Appropriate patient education is essential to boost patients’ trust and comprehension of biosimilars, hence lessening the influence of any nocebo effect.

Continuous education and communication initiatives are required to dismantle these obstacles and increase patient and physician confidence in biosimilars. This comprises precise and transparent information regarding the regulatory approval procedure, clinical equivalency and the possible advantages of biosimilars adoption.

### 6.3 Market dynamics and pricing strategies

Market dynamics, such as pricing schemes, reimbursement guidelines and competition are important factors in the uptake and economic consequences of biosimilars. Depending on a number of variables, including market competition, patent litigation and negotiations with payers or healthcare providers, the cost of biosimilars in comparison to originator biologics might differ significantly. In reaction to competition from biosimilars, originator biologics may lower their prices, diminishing thus the price difference and possibly the cost savings associated with biosimilars. Furthermore, price wars may result from the availability of several biosimilars for a single originator product, further complicating pricing strategies and market dynamics.

The adoption of biosimilars is further influenced by reimbursement rules. Payers may provide biosimilar users advantages in certain healthcare systems, such as lowered co-payments or preferential formulary placement. Reimbursement for biosimilars may be restricted or reduced in other systems, which would in turn decrease their uptake and economic impact. ^30^

### 6.4 Legal and patent issues

The adoption of biosimilars may also be hampered by legal and patent concerns. The potential for cost reductions can be diminished if biosimilar developers and originator biologic manufacturers engage in patent litigation that delays the introduction of biosimilars into the market. Furthermore, in order to prolong market exclusivity and delay the emergence of biosimilars, originator companies may employ patent “evergreening” tactics, whereby they seek new patents for minor alterations to their biologics. ^31^

## 7. Economic evaluation of biosimilars in RA

Economic evaluation studies confirm cost savings from the uptake of biosimilars in RA. In Poland, Stajszczyk et al. ^32^ used 12,687 infliximab, etanercept and adalimumab treatment courses to perform a retrospective budget impact analysis. Out of the estimated €243 million in overall savings for TNF inhibitors, €166 million were attributable to reduced treatment costs for rheumatoid and musculoskeletal diseases (RMDs) from the public payer perspective during an eight-year period. Real-world savings of €133 million and €107 million were estimated, respectively. Of the entire savings, the rheumatology sector generated between 68% and 92%. Overall, the mean annual cost of treatment dropped by 75% to 89%. Approximately 45,000 patients with RMDs may potentially be treated if all budget savings were used towards the reimbursement of extra TNF inhibitors.

In order to evaluate the budget impact of infliximab biosimilar CT-P13 in RA treatment during a three-year period in six Eastern European countries (Bulgaria, Czech Republic, Hungary, Poland, Romania and Slovakia), Brodszky et al. ^33^ built a model which considered only the direct drug costs in two scenarios. In the first scenario, 65% of patients started a new biologic therapy with CT-P13, and in the second, 80% switched from the originator to CT-P13. Taking into account a 25% discount over the originator price, estimated savings in the first scenario were €15.3 million, while savings in the second scenario were €20.8 million. In this model, 1,205 and 1,790 additional RA patients would receive therapy in the first and second scenarios, respectively, if budget savings were spent in the reimbursement of additional infliximab treatments.

A comparable budget impact model was created by Jha et al. ^34^ to assess CT-P13 in six immune-mediated diseases over a one-year period in five European countries: Germany, the UK, Italy, the Netherlands and Belgium. Based on a 25% switch rate and a 50% biosimilar uptake in newly-treated patients, the expected savings in RA for the 10%, 20% and 30% discount scenarios were €2.3, €4.6 and €6.9 million, respectively. According to this estimate, treating an extra 300, 676 and 1,158 RA patients would arise from the 10%, 20% and 30% discount scenarios, respectively.

In a real-world setting in France, Beck et al. ^35^ aimed at evaluating the potential cost savings after a year of using biosimilar infliximab to treat RA patients. Compared to adalimumab or etanercept, the cost of managing RA patients with biosimilar infliximab was significantly lower (€11,907 vs. €12,981 and €13,551 respectively). If every adult RA patient receiving treatment with infliximab changed to the biosimilar drug, the estimated annual cost savings would reach €13.6 million on a nationwide level. If all these savings were reallocated for the treatment of RA, 1,141 more patients could be treated.

The launch of infliximab and etanercept biosimilars resulted in savings of £38.8 million over a two-year period, according to an analysis of rheumatology specialties in the UK. ^36^ This was attributed to both the uptake of biosimilars and price reductions for originator products. Similarly, the availability of biosimilars in Scandinavian countries has heralded significant cost savings and increased patient access to biologics, despite already substantial use. ^37^

According to data from the Danish national DANBIO registry, ^38^ the etanercept biosimilar SB4 accounted for 84.2% of all etanercept use within a year of the originator etanercept patent expiration. When the first infliximab biosimilar was introduced in Denmark in 2015, a similar pattern was seen with infliximab biosimilars, with costs roughly reducing by two thirds when moving from originator to biosimilar infliximab. ^38^ According to another Danish study, after their market introduction, adalimumab biosimilars quickly replaced the originator drug. Overall, 95.1% of adalimumab prescriptions involved a biosimilar and thus, the cost of adalimumab treatments dropped by 82.8%. ^39^

Using hospital data from 2014 to 2018 in the Netherlands, Müskens et al. ^40^ examined how the introduction of biosimilars affected medication expenditure in RA patients and which patients acquired access to biologics due to the availability of biosimilars. They came to the conclusion that there was a consistent downward trend in the average cost per patient receiving a biologic treatment following the launch of biosimilars. However, as more patients began receiving biologic treatments, the budgetary relief brought about by the introduction of biosimilars to the market swiftly evaporated.

Valido et al. ^41^ examined efficacy, safety and cost savings of switching adult RA patients receiving originator infliximab for at least six months and with stable disease activity to biosimilar infliximab (CT-P13) in Portugal. Over a median follow-up duration of 15 months, disease activity remained steady and was comparable to the values noted with originator infliximab. The switch from originator infliximab to a biosimilar resulted in a 26.4% reduction in expenditure without compromising efficacy or safety. Switching RA patients from infliximab to the biosimilar CT-P13 in the UK, France and Germany would save €233 to €433 million over the course of five years (representing discounts of 20% and 30%, respectively). Over 7,500 more RA patients could receive biosimilar treatment thanks to the savings from the 30% discount. ^16^

For patients with established RA who do not respond to methotrexate, Patel et al. ^42^ compared two early intervention pathways (either adding originator biologic TNF inhibitors or adding biosimilar TNF inhibitors to methotrexate monotherapy at 6 months) with a standard intervention pathway (adding originator biologic TNF inhibitors to methotrexate monotherapy at 12 months). Comparing early intervention with an originator biologic TNF inhibitor at 6 months to standard intervention at 12 months, the early intervention group experienced increases in utilities of 0.10 quality-adjusted life-years (QALYs) per patient and total lifetime expenses of £1,692. An incremental cost-effectiveness ratio (ICER) of £17,335/QALY was obtained as a result. An early intervention with a biosimilar TNF inhibitor resulted in an ICER of £713/QALY, with costs rising by £70 and utilities by 0.10 QALYs per patient.

## 8. Discussion

Use of biosimilars can maximise benefits for RA patients by enhancing equity of access to biologics and providing affordable treatment for early disease control. Twelve strategies for the cost-effective use of bDMARDs were established through individual and group discussions by an international task force made up of thirteen specialists from seven European countries in the fields of pharmacology, epidemiology and rheumatology. ^43^ A Delphi procedure was used to develop a set of five overarching principles and twenty points to take into consideration for each strategy based on evidence from relevant systematic reviews and randomized controlled trials. Experts agreed that since approved biosimilars and originators are viewed similarly, all guidelines apply equally to both. Additionally, consensus was achieved that where a biosimilar is the most cost-effective version of the medication, it should be chosen if approved by a drug authority in a highly regulated area and that switching from an originator to one of its biosimilars should be taken into consideration if it lowers the overall cost of treatment. ^43^

The use of biosimilars in RA patients has drawn criticism in terms of economic implications. Evidence for the cost savings associated with biosimilars adoption in RA originates mostly from budget impact models. Nevertheless, this type of economic evaluation assesses the economic impact, does not target a beneficial investment, does not take into account health outcomes, considers the payer’s prospects, only looks at direct costs, has a short-term horizon and measures the total expenditure. Furthermore, because many of the variables (such as the official and real acquisition cost of biosimilar and originator drug, price discounts, the market share of biosimilars among newly treated and switching RA patients, treatment discontinuation or switching to other biologics, among others) are based on assumptions that may vary between regions, it is also challenging to predict the accuracy of budget impact models and the true extent of economic gains.

A central issue of the criticism pertaining to the economic implications of biosimilars use in RA is the originator-to-biosimilar non-medical switch (NMS) policies. NMS refers to changing a stable patient’s medication for reasons unrelated to medical necessity (usually driven by factors like cost, insurance coverage, or formulary changes, rather than the medication’s efficacy or safety). Real-world originator-to-biosimilar NMS studies have revealed a discontinuation rate for infliximab from 14.7% to 81.5% ^44^ and for etanercept from 7% to 17% ^45^ due to adverse events or loss of efficacy. A retrospective cohort analysis using the administrative databases of three local health units in Italy showed that the treatment costs for RA patients who switched their initial biologic medication during the first year of follow-up were higher than those who did not. ^46^ Gibofsky et al. ^47^ assessed the short-term costs of switching stable patients with autoimmune diseases from originator biologics to biosimilars in rheumatology from the perspective of US providers and third-party payers. For N=3,609 patients with an NMS rate of 16.6%, the total switching costs for payers were $771,460, primarily because of follow-up visits and extra laboratory testing. The primary cost driver in the sensitivity analyses was the NMS rate. The overall switching costs for payers increased to $1.19 and $2.39 million, when the NMS rate was raised to 25% and 50%, respectively. ^47^

The cost of NMS in a stable RA population (patients responding to originator etanercept with no treatment changes within the preceding 6 months) in the UK was estimated by Tarallo et al. ^48^ A survey of 150 rheumatologists from five EU countries (France, Germany, Italy, Spain, UK) provided data on the percentage of patients who switch therapies, baseline healthcare resource use and the effect of switching on resource use. According to this model, 1,259 (25.2%) of the 5,000 patients receiving originator etanercept would switch to a biosimilar. Following a three-month period, 26.3% of patients switched once more; 8.3% went back to the originator, 3.8% to a different biosimilar and 14.2% to a different biologic. In all impact scenarios, the switch was more expensive than continuing the originator’s treatment, even if originator etanercept cost more than biosimilars. Annual costs per patient were higher when switching treatments than when staying on the originator treatment. Switching was associated with higher utilization of healthcare resources. ^48^

In the short term, any additional resources needed to support the switch from an originator product to a biosimilar - such as extra outpatient appointments – may potentially offset the biosimilar’s cost savings. Although data from the DANBIO registry showed no evidence of an increase in outpatient visits following a switch from originator infliximab to a biosimilar, ^49– 51^ Moorkens et al. ^52^ found that in some Swedish counties, the extra effort required to actively swift patients from the originator product did not outweigh the slight cost reduction provided by biosimilar etanercept.

Whether or not the use of NMS in patients with stable RA results in higher costs -measured not only on adverse events and loss of efficacy, but also on hospitalization costs, missed workdays, absenteeism and worsening QALY with an increase in ICER over time – will be confirmed by real-world data and cost-effectiveness analyses. Future cost-effectiveness evaluations must, therefore, make every effort to be as precise as possible, to include the most relevant data and be updated on a regular basis.

## 9. Conclusions

The adoption of biosimilars among RA patients has a major economic impact and important consequences for the sustainability of healthcare systems. Biosimilars represent a viable substitute for expensive biologics, allowing healthcare systems to lower RA-related costs, enhance patient access to biologic treatments and reallocate savings to other patient care aspects.

The prices of originator products have gradually decreased as a result of significant price differences that exist at launch between biosimilars and originators. Consequently, biosimilars offer an opportunity to increase competition and save costs for healthcare systems. In fact, budget impact assessments have indicated that the adoption of biosimilars may result in significant cost savings. The possible budget increases from expanding access to biologics and offering additional services to RA patients must be weighed against such cost savings.

But in order for biosimilars to be successfully adopted, concerns with legal and patent matters, market dynamics, physician and patient acceptability and regulatory issues must be addressed. Healthcare systems can optimize the economic benefits of biosimilars and strengthen their sustainability amidst escalating healthcare expenditures by enacting supportive policies, including the engagement of relevant stakeholders and the generation of real-world evidence. It is envisaged that the broader adoption of biosimilars will help to lessen the disparities in biologic treatment utilization and potentially reduce the financial and social burden of RA.

## 10. Future directions and recommendations

In order to optimize the economic benefits of biosimilars for the sustainability of healthcare systems, a number of approaches and recommendations may be worthwhile considering. To assist in the effective incorporation of biosimilars into clinical practice, these include policy initiatives, stakeholder engagement and ongoing research and monitoring.

### 10.1 Policy initiatives

Policymakers are an important factor in influencing the climate for the acceptance of biosimilars. The adoption of biosimilars and their economic impact can be increased by putting in place rules that support competition, guarantee fair pricing and encourage their use. Policies that, for instance, promote price transparency, introduce less obstacles to market entry and assist in the development of biosimilars can all help create more sustainable and competitive healthcare systems. Policies for reimbursement should also support the use of biosimilars and lower healthcare expenditure. Some examples can include reduced co-payments for RA patients, preferential reimbursement for biosimilars and value-based pricing schemes that take into account the economic and clinical benefits of biosimilars. ^53^

### 10.2 Stakeholder engagement

Building trust in biosimilars and guaranteeing their effective adoption requires the engagement of important stakeholders, such as payers, patients and healthcare professionals. In addition to addressing safety, effectiveness and immunogenicity issues, education and communication campaigns should emphasize the potential economic benefits of biosimilars and their contribution to the sustainability of healthcare systems.

In the context of switching to biosimilars, informed shared decision-making between patients and healthcare providers is crucial. Patients need to be made aware of the benefits of switching, the evidence in favor of using biosimilars and how switching might affect how well they respond to treatment. This may allay worries and encourage patients to adhere to their biosimilar treatment. In order to obtain savings in healthcare budgets, policymakers should ensure significant price reductions and encourage physicians to embrace biosimilar products. ^54^

### 10.3 Ongoing research and monitoring

Ongoing research and monitoring are essential for guaranteeing the long-term safety, efficacy and economic impact of biosimilars. Data on patient outcomes, adherence to therapy and use of healthcare resources are examples of real-world evidence from clinical practice that can offer important insight into how well biosimilars work in various contexts.

In order to monitor the immunogenicity and safety of biosimilars, pharmacovigilance activities should be stepped up, especially as more patients are switched from originator biologics. This entails putting in place reliable surveillance systems to monitor side effects and long-term results in RA patients who are treated with biosimilars.

Lastly, economic analyses of the adoption of biosimilars ought to keep evaluating their cost-effectiveness, budget impact and wider implications for the sustainability of healthcare systems. The best possible use of biosimilars in clinical practice can be supported by the ability of these evaluations to inform policy decisions, pricing strategies and reimbursement models. ^55^

## Author contributions

CN and NK led the conception and design of the study; CN, JF and MT conducted the literature search and synthesized the information from the selected studies; CN drafted the manuscript; NK, JF and MT helped to critically revise the manuscript. All authors read and approved the final manuscript.

## Data Availability

No data are associated with this article.
