# Supplementary material for: Fostering healthcare system sustainability through efficient practices: Can adopting biosimilars ease the financial burden of rheumatoid arthritis?
Source: F1000Res. 2025 Apr 4;13:1128. Originally published 2024 Oct 4. [Version 2] doi: 10.12688/f1000research.156983.2 (PMC11983381; doi:10.12688/f1000research.156983.2)
Supplement: Peer review history

# Reviewer report: Reviewer response for version 2

**DOI:** 10.5256/f1000research.179913.r375569

**Published:** 2025-04-08

**Authors:** George Bertsias

**Affiliations:** 1 Rheumatology, Clinical Immunology and Allergy Department, University of Crete School of Medicine, Heraklion, Greece; 2 Laboratory of Autoimmunity-Inflammation, Institute of Molecular Biology and Biotechnology, Heraklion, Greece

I am satisfied with the revised version.

Is the review written in accessible language?

Yes

Are all factual statements correct and adequately supported by citations?

Yes

Are the conclusions drawn appropriate in the context of the current research literature?

Yes

Is the topic of the review discussed comprehensively in the context of the current literature?

Yes

Reviewer Expertise:

Autoimmune rheumatic diseases, lupus, outcomes research, genomics

I confirm that I have read this submission and believe that I have an appropriate level of expertise to confirm that it is of an acceptable scientific standard.

# Reviewer report: Reviewer response for version 2

**DOI:** 10.5256/f1000research.179913.r375570

**Published:** 2025-04-08

**Authors:** Georgios Filippou

**Affiliations:** 1 University of Milan, Milan, Italy

My concerns have been adequately addressed. I have no further comments.

Is the review written in accessible language?

Yes

Are all factual statements correct and adequately supported by citations?

Partly

Are the conclusions drawn appropriate in the context of the current research literature?

Yes

Is the topic of the review discussed comprehensively in the context of the current literature?

Yes

Reviewer Expertise:

Rheumatology

I confirm that I have read this submission and believe that I have an appropriate level of expertise to confirm that it is of an acceptable scientific standard.

# Reviewer report: Reviewer response for version 1

**DOI:** 10.5256/f1000research.172375.r367677

**Published:** 2025-03-25

**Authors:** George Bertsias

**Affiliations:** 1 Rheumatology, Clinical Immunology and Allergy Department, University of Crete School of Medicine, Heraklion, Greece; 2 Laboratory of Autoimmunity-Inflammation, Institute of Molecular Biology and Biotechnology, Heraklion, Greece

This is well-structured, comprehensive analysis of the implications of biosimilars use in rheumatoid arthritis. My comments are as follows: a) adopting biosimilars holds potential to reduce the financial costs related to the exposure to biological agents and thus, indirectly led to improved patient outcomes (due to wider/earlier institution of such drugs).

 This should be made explicit in the text to avoid misinterpretation; b) a short discussion about targeted synthetic DMARDs (eg JAK inhibitors) and the prospect of having biosimilars may worth to be added; c) a table summarizing the findings from budget analysis studies with biosimilars presented in the main text may help.

Is the review written in accessible language?

Yes

Are all factual statements correct and adequately supported by citations?

Yes

Are the conclusions drawn appropriate in the context of the current research literature?

Yes

Is the topic of the review discussed comprehensively in the context of the current literature?

Yes

Reviewer Expertise:

Autoimmune rheumatic diseases, lupus, outcomes research, genomics

I confirm that I have read this submission and believe that I have an appropriate level of expertise to confirm that it is of an acceptable scientific standard.

# Response

**Published:** 2025-03-25

**Authors:** Christos Ntais

**Affiliations:** Open University of Cyprus, Cyprus

We thank the Reviewer for giving us the opportunity to improve our work. Please find our feedback on your comments below. 

 1. We have added the following text (Subsection 5.3): In other words, the adoption of biosimilars in the treatment of rheumatoid arthritis not only reduces the financial costs associated with biological agents but also has the potential to indirectly improve patient outcomes. By making these treatments more affordable, biosimilars can facilitate earlier and broader access to biologic therapies, which may lead to improved long-term health outcomes for patients. This expanded access is particularly crucial in optimizing disease management and minimizing the long-term effects of RA.

 2. We have added the following Subsection (10.4): The potential for biosimilars of JAK inhibitors. In addition to bDMARDs, targeted synthetic DMARDs (tsDMARDs), such as Janus kinase (JAK) inhibitors, have emerged as important treatment options for RA. These therapies offer oral alternatives to biologics, providing flexibility in treatment management. The potential development of biosimilars for JAK inhibitors could further enhance access to these therapies, potentially reducing treatment costs while maintaining therapeutic efficacy. As with biosimilars in bDMARDs, the introduction of biosimilars for tsDMARDs may improve patient outcomes by enabling earlier and broader access to effective treatments, contributing to better disease control and long-term health benefits.

 3. We appreciate the importance of presenting data in a clear and accessible format. However, we believe that adding such a table may be redundant, as all the relevant budget analysis findings are already comprehensively discussed within the main text. The current structure ensures that the financial implications of biosimilar adoption are integrated into the broader discussion, maintaining the logical flow of the manuscript.

# Reviewer report: Reviewer response for version 1

**DOI:** 10.5256/f1000research.172375.r364259

**Published:** 2025-03-18

**Authors:** Georgios Filippou

**Affiliations:** 1 University of Milan, Milan, Italy

- The paper presents a comprehensive and well-researched examination of biosimilars in rheumatoid arthritis treatment. It effectively integrates recent findings, making it a valuable contribution to the ongoing discussion on biologic therapies in rheumatology.
- The topic is timely and relevant, given the increasing utilization of biosimilars in various healthcare systems and the implications for treatment accessibility and cost-effectiveness.
- The paper demonstrates a good review of existing recent literature that addresses the management of RA with biosimilars. The authors adopt a comprehensive approach by considering clinical, economic and societal perspectives, which is essential for understanding the complexities of biosimilar adoption and usage in different healthcare contexts.
- Studies from various countries are included, which enhances the paper's relevance. The paper highlights the importance of patient access to biosimilars and the economic burden of RA treatments.

To enhance clarity and applicability, I suggest the following revisions:

-   The authors could further enhance the discussion by incorporating more qualitative studies on patient experiences and attitudes toward biosimilars, in terms of acceptance and adherence.
-   Adding case studies from countries or healthcare systems that have successfully integrated biosimilars for rheumatoid arthritis treatmen could strengthen the practical applicability of the findings.

With these revisions, the paper has the potential to make a significant contribution to the literature and serve as a valuable resource for both clinicians and policymakers.

Is the review written in accessible language?

Yes

Are all factual statements correct and adequately supported by citations?

Partly

Are the conclusions drawn appropriate in the context of the current research literature?

Yes

Is the topic of the review discussed comprehensively in the context of the current literature?

Yes

Reviewer Expertise:

Rheumatology

I confirm that I have read this submission and believe that I have an appropriate level of expertise to confirm that it is of an acceptable scientific standard, however I have significant reservations, as outlined above.

# Response

**Published:** 2025-03-25

**Authors:** Christos Ntais

**Affiliations:** Open University of Cyprus, Cyprus

We thank the Reviewer for giving us the opportunity to improve our work. Please find our feedback on your comments below.

 1. The following paragraph has been added (Subsection 6.2): Several qualitative studies have explored patient experiences and attitudes toward biosimilars, focusing on factors influencing acceptance and adherence. Chew et al. ^30^ examined patient experiences before and after switching to biosimilars under British Columbia’s mandatory policy. Participants initially expressed apprehension about efficacy and safety but reported successful transitions with support from healthcare providers and family. The study underscores the importance of effective communication and support systems in facilitating biosimilar acceptance. Similarly, Ryan et al. ^31^ assessed patient views on switching to biosimilars for inflammatory bowel disease within the Veterans Health Administration. Findings revealed that patients perceived biosimilar switching as riskier than switching to generic medications. Key themes included concerns about risks and benefits, the importance of patient-provider communication, shared decision-making and individualized care. Finally, Rosembert et al. ^32^ explored the perspectives of patients and consultants regarding the transition from originator biologics to biosimilars. It emphasized the necessity of effective communication, timely information dissemination and the potential role of pharmacists in facilitating the switching process. The study also highlighted the importance of addressing patient concerns to ensure successful transitions.

 2. We appreciate the suggestion to include additional case studies from countries or healthcare systems that have successfully integrated biosimilars into RA treatment. However, the manuscript already contains several case studies from a variety of countries that effectively demonstrate the feasibility, cost-effectiveness and real-world application of biosimilars in RA treatment. These examples were selected to provide a comprehensive view of how biosimilars have been successfully integrated into different healthcare systems worldwide. We believe that adding further case studies at this point could introduce redundancy without substantially enhancing the manuscript’s overall contribution, as the current case studies already cover the key aspects of biosimilar adoption in diverse contexts.
